# Giant Posterior Fossa Neurocryptococcoma Mimicking a Brain Tumor in an Immunocompetent Patient: A Case Report and Literature Review

**DOI:** 10.7759/cureus.79284

**Published:** 2025-02-19

**Authors:** Valdecir B Spenazato Junior, Enzo L Campos, Gabriel B Petronilho, Ricardo Santos de Oliveira, Matheus F. M Ballestero

**Affiliations:** 1 Department of Neurological Surgery, Fundação Pio XII, Barretos, BRA; 2 Department of Medicine, Federal University of Jataí, Jataí, BRA; 3 Department of Medicine, Centro Universitário Fundação Assis Gurgacz, Cascavel, BRA; 4 Division of Neurosurgery, Department of Surgery and Anatomy, Ribeirão Preto Clinics Hospital, Ribeirão Preto Medical School, University of São Paulo, Ribeirão Preto, BRA; 5 Department of Surgery and Anatomy, University Hospital of Ribeirão Preto Medical School, University of São Paulo, Ribeirão Preto, BRA; 6 Department of Medicine, Federal University of São Carlos, São Carlos, BRA

**Keywords:** brain neoplasms, central nervous system, cryptococcosis, cryptococcus, infratentorial neoplasms

## Abstract

Cryptococcosis is a rare infection that can affect the central nervous system (CNS), leading to neurological complications. Neurocryptococcoma, though uncommon, can present with significant morbidity. We report the case of a 31-year-old immunocompetent woman who developed a large posterior fossa neurocryptococcoma. The lesion was successfully treated with complete surgical resection via occipital craniotomy. Diagnosing a cryptococcal infection can be challenging due to its rarity and the variability of its presentation. Management typically involves either medical or surgical interventions based on lesion characteristics. This case highlights the importance of considering neurocryptococcoma in the differential diagnosis for patients with neurological symptoms, even in those without apparent immune deficiency.

## Introduction

Cryptococcosis is an opportunistic disease more commonly found in immunocompromised patients, such as HIV-infected individuals, patients with hematological malignancies, solid-organ transplant recipients, and those undergoing immunosuppressive therapy. An estimated 223,100 individuals develop cryptococcal meningitis annually, resulting in 181,100 deaths and accounting for 15% of AIDS-related mortality. The disease is caused by *Cryptococcus neoformans*, an organism that can infect both immunocompromised and immunocompetent individuals by entering the human body through the respiratory tract as environmental propagules, subsequently spreading hematogenously from the lungs to the central nervous system (CNS) [1-4].

Intracranial cryptococcosis typically presents with headache, fever, and nuchal stiffness, but in some cases, it may cause a sizable, space-occupying brain lesion, making it the most common type of intracranial fungal mass lesion, with symptoms varying according to its location. The radiologic features of classical cryptococcosis are distinctive, occurring almost exclusively in the basal ganglia due to the contiguous spread of the organism from basal meningitis through the Virchow-Robin perivascular spaces, presenting as a ring-enhancing lesion typically ranging from 3 to 10 mm in diameter, though in rare cases it may evolve into a significantly sized chronic granuloma [2,5].

According to Akins and Jian, the clinical evolution leading to cryptococcoma begins with the inhalation of *Cryptococcus* species (sp.), which colonizes the nasal regions and later disseminates to the lungs, brain, and muscles [6]. After the fungal infection, some patients, particularly immunocompetent individuals without underlying diseases, may remain asymptomatic or present with subclinical disease. The development of cryptococcosis depends on the correlation between the host, exposure, and the cellular immunity of the infected patient. In a smaller number of patients, cryptococcosis may progress to the central nervous system and present itself in a granulomatous form as an intraparenchymal expansive lesion that mimics brain neoplasms.

This paper presents a rare case of a giant posterior fossa neurocryptococcoma, along with a literature review, and includes a surgical video to enhance the reader's understanding.

## Case presentation

**Figure 1:**
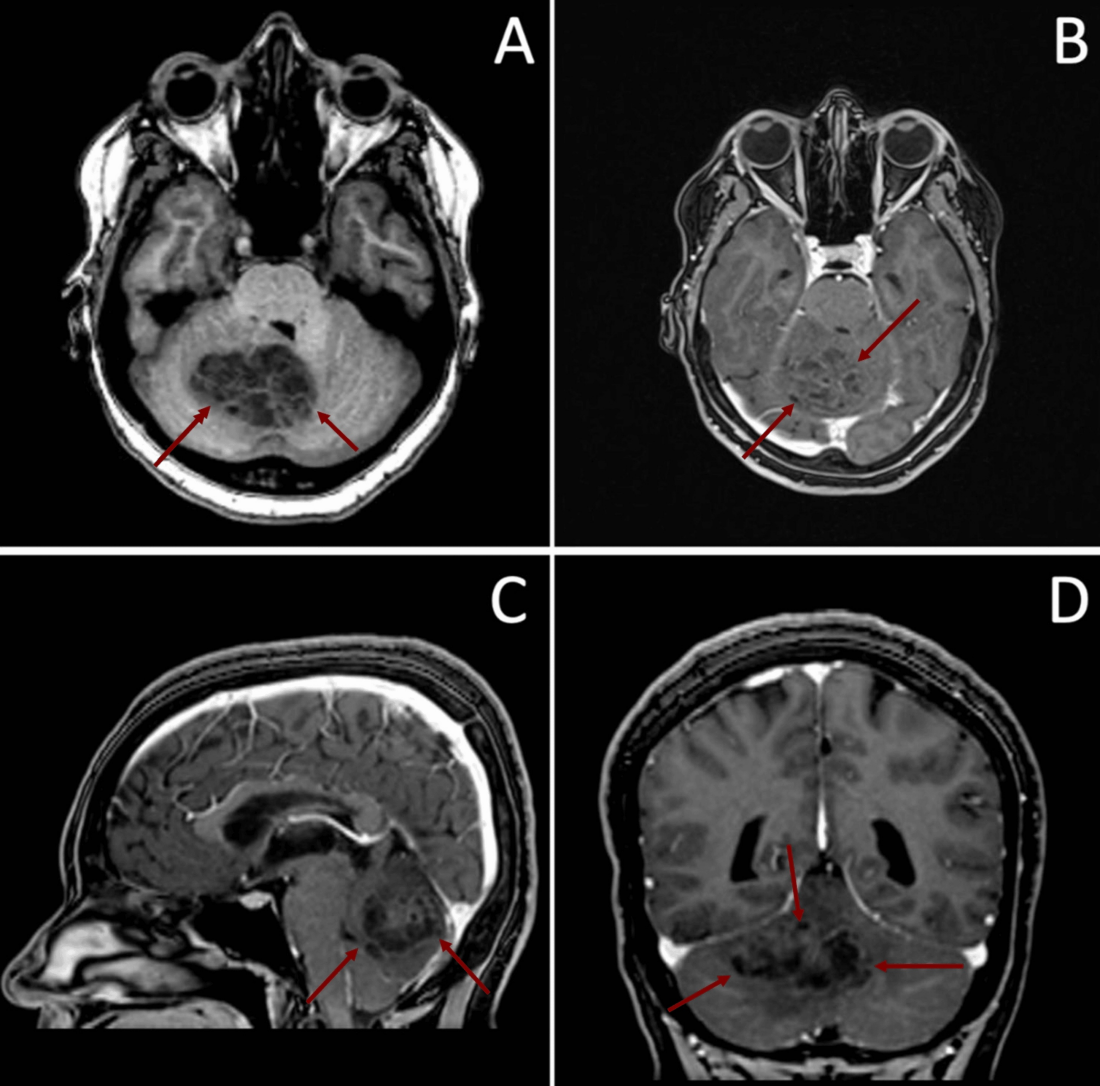
(A) Axial T1-weighted MRI showing heterogeneous space-occupying lesion on posterior fossa (red arrows); (B) axial post-gadolinium T1-weighted MRI showing slight post-contrast reinforcement (red arrows); (C) sagittal contrast-enhanced MRI showing slight post-contrast reinforcement (red arrows); (D) coronal contrast-enhanced MRI showing heterogeneous space-occupying lesion on posterior fossa (red arrows). MRI: magnetic resonance imaging

A 31-year-old woman presented with symptoms of headache, vertigo, and walking imbalance for 30 days before admission. On physical examination, she had gait ataxia and right appendicular dysmetria, without any cranial nerve alterations. Brain magnetic resonance imaging (MRI) was performed using T1-weighted sequences both with and without contrast enhancement, which revealed a heterogeneous cerebellar lesion with slight T1 post-contrast enhancement posterior to the fourth ventricle, with a diameter of approximately 4 cm at its largest point (Figure 1). MRI characteristics, as described in the image, do not allow differentiation between neoplasms and fungal lesions. Investigation for a possible primary tumor site in other organs, using thoracoabdominal tomography, did not reveal any lesions.

**Figure 2:**
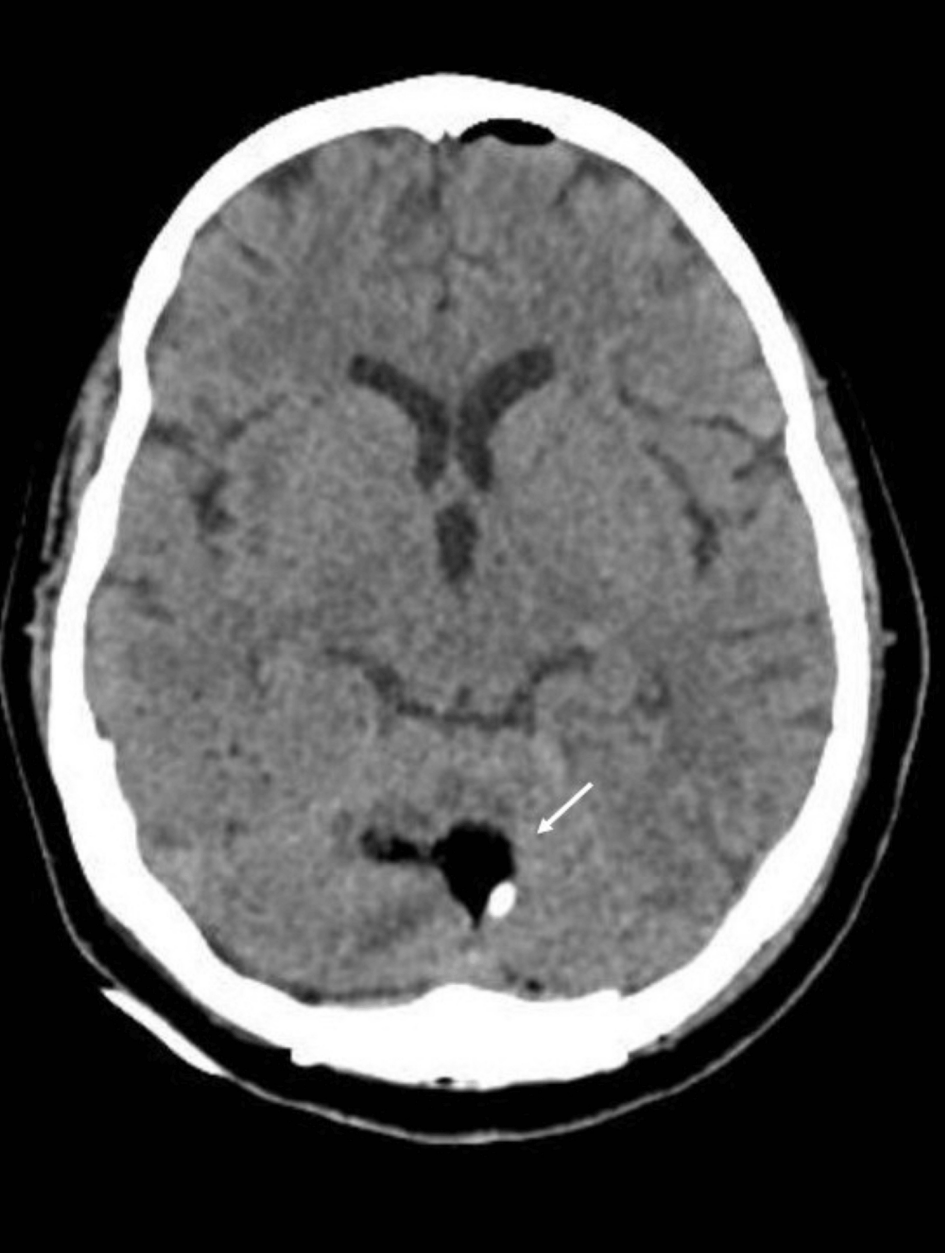
Postoperative axial non-contrasted CT showing the operative site (white arrow). CT: computed tomography

**Figure 3:**
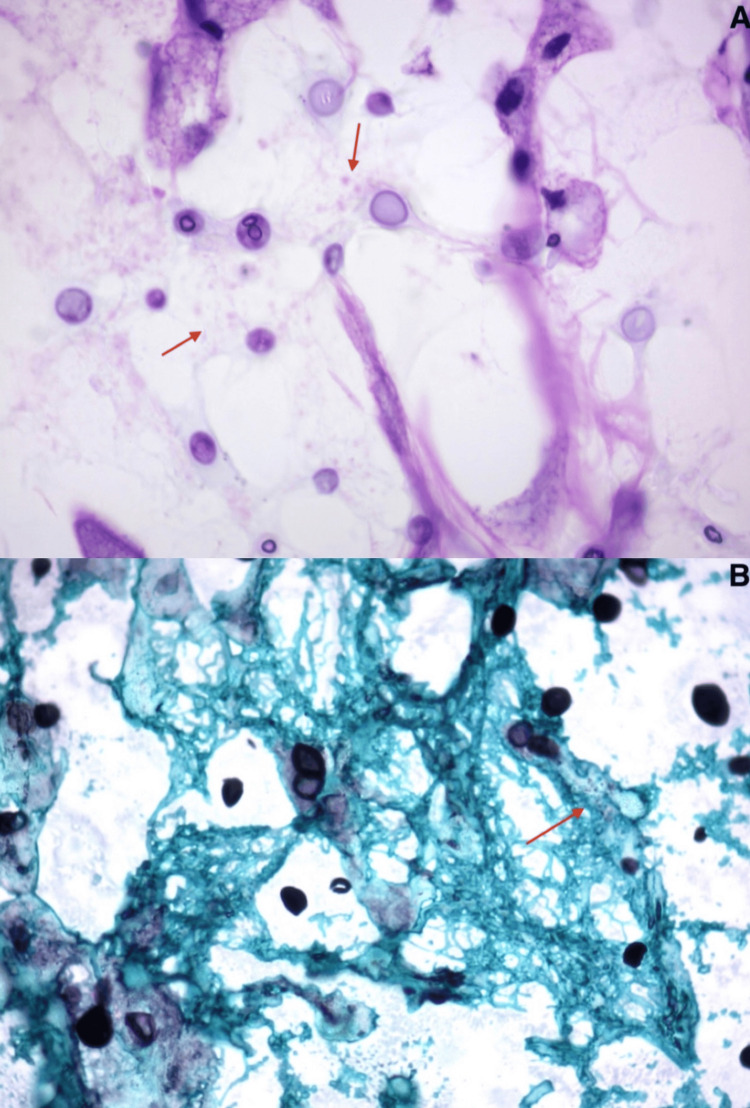
Histopathological results. (A) H&E stain. (B) Grocott-Gomori's methenamine silver staining. Arrows point to *Cryptococcus*.

The patient underwent complete surgical resection via occipital craniotomy with direct access to the lesion (Figure 2 and Video 1). The day following the surgery, the patient was stable with no new neurological deficits, and the immediate postoperative computed tomography (CT) was unremarkable (Figure 3).

**Video 1:** Neurocryptococcoma resection.

**Figure 4:**
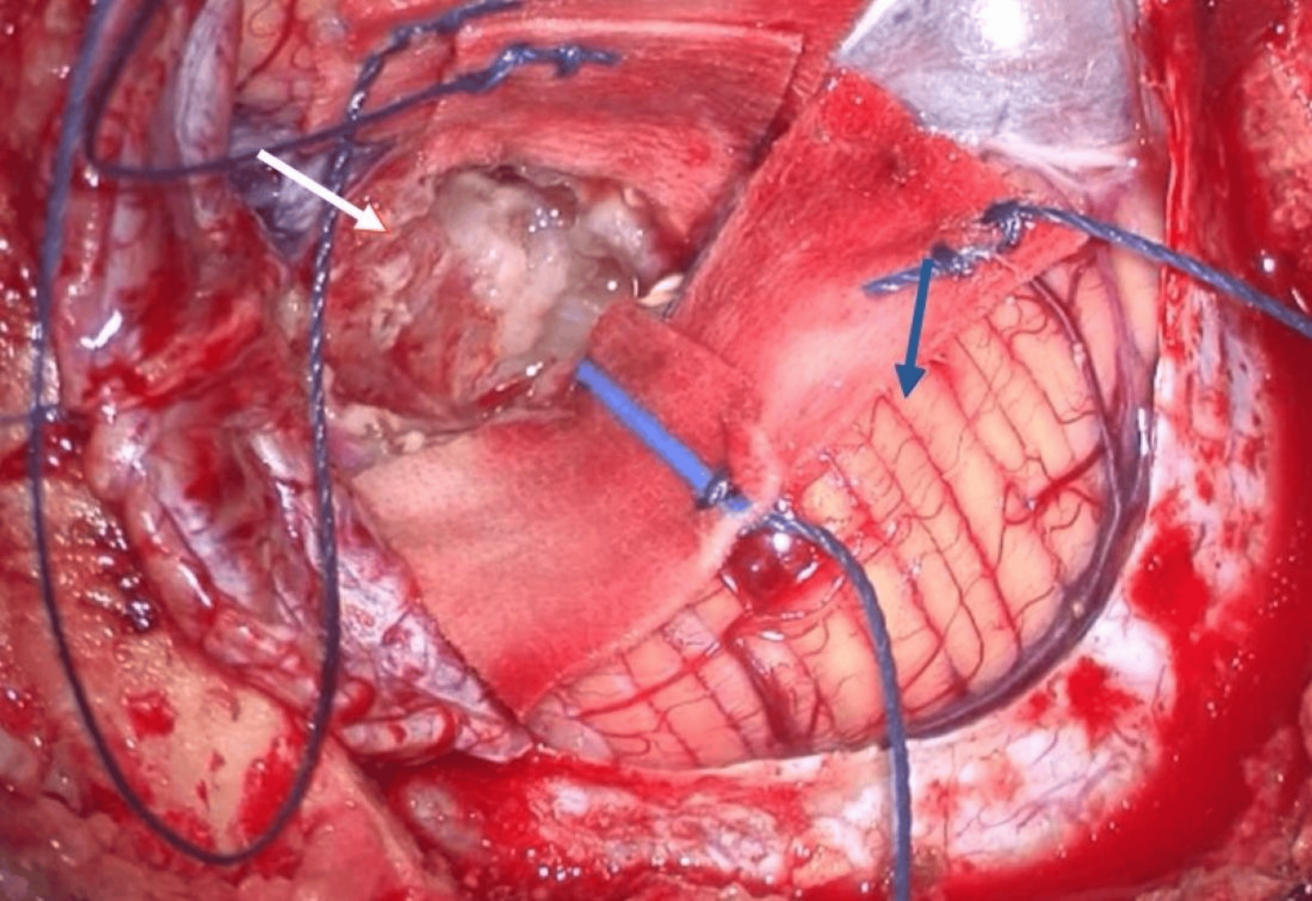
Intraoperative aspect of the neurocryptococcoma after initial lesion debulking (blue arrow, normal cerebellum; white arrow, neurocryptococcoma).

However, on the seventh postoperative day, the patient developed a cerebrospinal fluid (CSF) leak, hydrocephalus, and sepsis, with rapid progression to death. Screening for dissemination to other parts of the body was not performed because the pathological report was not available at the time. CSF examination revealed a positive India ink test, and the subsequent pathological analysis of the lesion confirmed an organized neurocryptococcoma (Figure 4).

## Discussion

Massive fungal lesions in the central nervous system that are sufficient to cause neurological deterioration are extremely rare, but in immunocompromised patients, they must be considered in the differential diagnosis. Cryptococcosis, caused by *Cryptococcus* species (sp.) such as *Cryptococcus neoformans* and *Cryptococcus gattii*, is the main cause of disseminated CNS infections [7]. The course of the infection is determined by the host's immune system, and immunocompromised patients may present with atypical manifestations, such as pulmonary cryptococcosis with hematoencephalic dissemination, punctate lesions, perivascular deposits, and nodules with a ringlike appearance. In contrast, cryptococcoma formation can also occur in immunocompetent patients and can mimic neoplasms [8]. However, primary and secondary brain tumors are usually the initial differential diagnoses in these cases. The clinical presentation of cryptococcosis is nonspecific and may include meningitis, headache, seizures, fever, altered mental status, and/or pyramidal symptoms, depending on the location of the lesions [2].

Similar to CNS neoplasms, neurocryptococcomas have been reported previously in immunocompetent patients, with the interventricular region being the most common location [5,9], contradicting earlier reports by Akins and Jian [6]. In a case described by Santander et al., a patient presented with hydrocephalus, which was diagnosed as a fungal infection after undergoing a third ventriculostomy by endoscopy, thus highlighting the importance of considering cryptococcosis as a differential diagnosis in CNS pathologies [10].

*Cryptococcus neoformans* is a neurotropic pathogen with global dissemination and is restricted to the respiratory system and CNS. On the contrary, *Cryptococcus gattii* has its progression to cryptococcoma in the CNS of HIV-positive patients; therefore, research should always be carried out in patients positive for the infection [6]. Furthermore, it presents greater dissemination in tropical areas leading to the conditions already described.

*Cryptococcus* is a neurotropic pathogen that can cause respiratory and central nervous system (CNS) infections. *Cryptococcus neoformans* is the most common cause of disseminated CNS infections, while *Cryptococcus gattii* is more commonly associated with CNS cryptococcoma in HIV-positive patients [6]. Studies should therefore focus on patients with confirmed *Cryptococcus* infection. In addition, *Cryptococcus gattii* has been found to have greater dissemination in tropical areas, which may contribute to the conditions already described.

The treatment of neurocryptococcosis requires a specific diagnosis and the exclusion of differential diagnoses. Therefore, a combination of complementary examinations, including magnetic resonance imaging (MRI), cerebrospinal fluid puncture, culture, and spectroscopy, is crucial. Although imaging is important, histological examination remains the gold standard for confirmation [11].

In a study by Duarte et al., MRI was analyzed in T1 and T2 with confirmed microbiology of neurocryptococcosis in 19 patients with competent and suppressed immunity [12]. The topography of the lesion was evaluated, and the alterations found included leptomeningeal and pachymeningeal enhancement, the involvement of the perivascular space (dilations), *Cryptococcus* granulomas, hydrocephalus, miliary nodules, and plexitis. Immunosuppressed patients exhibited miliary dissemination, while immunocompetent patients showed granulomas of variable diameters in T1, in low intensity, and T2, in high intensity [13]. Chronic patients manifested vasogenic conditions.

In order to better visualize brain structural changes, the use of contrast with doubled dosage in immunocompromised patients (20 mL of gadolinium) is recommended. Tan et al. [1] analyzed 18 immunocompetent patients with identical results to those reported by Duarte et al. [12] but with involvement of the basal nuclei. Although MRI findings lack specificity and do not reveal any immunological and/or etiological relationships, the ring-shaped enhancement of the mass lesion with or without cystic changes on MRI may suggest cryptococcoma, and the definitive diagnosis depends on the anatomopathological study of the lesion specimen [14].

Meningitis is the most prevalent clinical finding in the literature, but it can be absent in some cases, leading to a false negative cryptococcal latex antigen agglutination test. Therefore, it should still be considered a differential diagnosis, particularly in immunosuppressed patients, which may indicate the need for further testing [11]. In immunocompromised patients, the clinical presentation is often nonspecific, with symptoms such as headache, fever, convulsions, and altered consciousness. Imaging findings on CT and MRI may include ventricular dilation, leptomeningeal enhancement, and infarcts of the Virchow-Robin spaces, which are visible on MRI. In some cases, there may be no findings at all [13].

In a report by Hospenthal and Bennett, an immunocompetent patient with CNS cryptococcoma was diagnosed and initially treated as a demyelinating disease, highlighting the diagnostic challenges associated with CNS cryptococcoma [15]. Clinicians should be aware of the not infrequent sequelae that persist in these patients. The persistence of lesions on neuroimaging should not be misinterpreted as evidence of active cryptococcosis. Additionally, the recurrence of surgically removed cryptococcoma may occur, as described in a patient with granulomatous cryptococcoma who did not adhere to antifungal drug therapy and did not receive proper follow-up care [16].

Treatment

According to Santander et al., early treatment with antifungal agents has been shown to be effective in patients with neurocryptococcosis [10]. While corticosteroids may reduce edema in some patients, there is insufficient clinical evidence to support their routine use in this setting. Treatment with antifungal agents alone may not be sufficient, particularly for large lesions, as complete remission is often not achieved [6,13].

Combination therapy with antifungal agents and emergency measures may improve prognosis [6]. Lumbar punctures can be used to manage intracranial pressure, as well as hyperosmolar therapy, external ventricular drainage (EVD), and ventriculoperitoneal (VP) shunt. Despite these interventions, the prognosis for neurocryptococcosis is generally poor.

**Figure 5:**
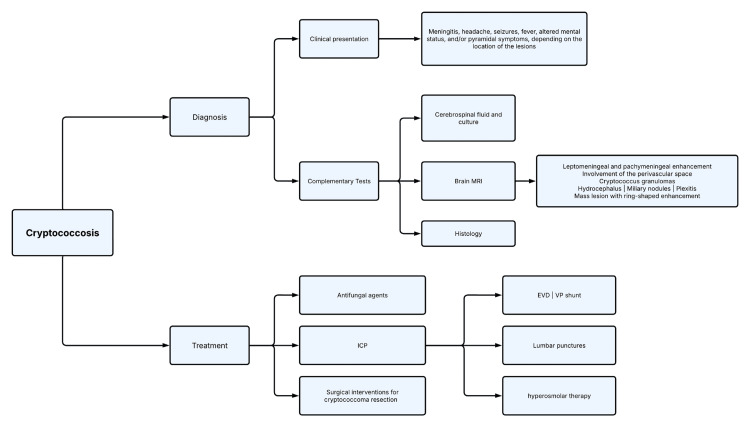
Cryptococcosis: diagnostic approach and treatment flowchart. This flowchart summarizes the key elements for the cryptococcosis diagnosis and treatment. MRI, magnetic resonance imaging; ICP, intracranial pressure; EVD, external ventricular drainage; VP, ventriculoperitoneal

It is important to note that patients with neurocryptococcosis may experience a recurrence of surgically removed cryptococcoma if they do not adhere to antifungal drug therapy and regular follow-up. Thus, strict adherence and close monitoring are crucial for the successful management of this condition (Figure 5) [15].

## Conclusions

Massive fungal lesions in the central nervous system (CNS) are rare but should always be considered in immunocompromised patients due to their increased susceptibility to disseminated infections, including cryptococcosis. The clinical presentation is often nonspecific, with symptoms such as headache, seizures, fever, altered mental status, and pyramidal signs, making a broad differential diagnosis necessary. Imaging studies, particularly MRI, can reveal characteristic findings such as leptomeningeal and pachymeningeal enhancement, perivascular space involvement, granulomas, hydrocephalus, miliary nodules, plexitis, and ring-enhancing mass lesions. Although imaging and complementary tests, including cerebrospinal fluid (CSF) analysis, culture, and spectroscopy, are valuable, histological examination remains the gold standard for a definitive diagnosis. Treatment primarily consists of antifungal therapy, but some cases may require adjunct interventions such as repeated lumbar punctures, hyperosmolar therapy, external ventricular drainage (EVD), ventriculoperitoneal (VP) shunt placement or surgical management to control intracranial pressure and address complications. As illustrated in the case, managing the infection can be difficult, and complications such as dissemination, sepsis, recurrence, and death can occur, regardless of the chosen treatment.
